# Mild cognitive impairment in Parkinson’s disease: a distinct clinical entity?

**DOI:** 10.1186/s40035-017-0094-4

**Published:** 2017-09-13

**Authors:** Ming-Ching Wen, Ling Ling Chan, Louis C.S. Tan, Eng King Tan

**Affiliations:** 1 Department of Research, National Neuroscience Institute, Singapore, Singapore; 2 Department of Neurology, National Neuroscience Institute, 11 Jalan Tan Tock Seng, Singapore, 308433 Singapore; 3 Department of Diagnostic Radiology, Singapore General Hospital, Singapore, Singapore; 4 Duke-NUS Medical School, Singapore, Singapore

**Keywords:** Mild cognitive impairment, Parkinson’s disease, Alzheimer disease, Lewy body, Neuropsychiatric comorbidities, Cognitive reserve

## Abstract

**Background:**

Mild cognitive impairment in Parkinson’s disease (PD-MCI) is a common clinical condition. Understanding its pathology and clinical features is important for early intervention before the onset of dementia. In the past, variable definitions and differences in neuropsychological batteries generated divergent results of the affected cognitive patterns.

**Main body:**

The introduction of PD-MCI criteria by the Movement Disorders Society (MDS) Task Force provides a more uniform system for defining and measuring PD-MCI and may improve the validity of future research. PD-MCI is likely to be heterogeneous since it can coexist with Alzheimer’s disease and/ or Lewy body pathologies in PD. Pathogeneses of neuropsychiatric disturbances, such as depression, anxiety and apathy, are associated with PD with or without MCI. In addition, cognitive reserve formed by patients’ unique life experiences may influence the outward cognitive performance despite the presence of the aforementioned pathogeneses and hence alter the diagnosis of MCI.

**Conclusion:**

The overlap of cognitive impairment across different neurodegenerative diseases suggests that PD-MCI is likely to result from a mixture of complex pathophysiologies, rather than being a distinct pathologic entity. Differentiating MCI from other organic symptoms in PD would facilitate novel therapeutic strategies.

## Background

Parkinson’s disease (PD) is one of the most common neurodegenerative diseases. Although it is primarily a motor disorder, accumulating evidence has suggested non-motor symptoms, such as cognitive impairment, can significantly impact health-related quality of life and even prognosis [1, 2]. Mild cognitive impairment (MCI) refers to an intermediate phase between normality and dementia, with both subjective and objective cognitive impairment but with preserved activities of daily living [3]. Identification of this intermediate phase is of much importance because early intervention may be more effective before the disease progresses [4].

The prevalence of MCI (PD-MCI) in PD ranges from 15% to 53% across studies as shown in a previous review by Yarnall et al. [5]. The large variation in the prevalence of patients with PD-MCI is likely confounded by different sources of participants (community- versus hospital-based), variable demographic and clinical characteristics and importantly, diverse definitions of PD-MCI [5]. To overcome the problem of inconsistent definitions of PD-MCI in the research field, in 2012, the Movement Disorders Society (MDS) commissioned a taskforce to unify the diagnostic criteria for PD-MCI based on a literature review and expert consensus [6].

### Cognitive manifestations of PD-MCI

While the effort of the MDS has improved the clarity of PD-MCI, the entity is likely to be heterogeneous [7]. It can be further divided into single-domain and multiple-domain subtypes; each of which may show impairment in amnestic or non-amnestic (e.g., executive or visuospatial dysfunction) domain [8, 9]. Numerous studies have attempted to elucidate the most common MCI subtypes and their associations with later development of dementia in PD. Although patients with PD-MCI often suffer from impairment in a range of cognitive domains, such as executive function, attention, processing speed, visuospatial ability, learning and memory, most patients present the non-amnestic subtype [10–12]. Compared to the predominance of memory impairment in MCI due to Alzheimer’s disease (AD), cognitive deficits associated with PD-MCI tend to be non-amnestic [11, 12] and may involve greater frontal-based dysfunctions, including executive and attention/ working memory deficits [7, 13, 14]. However, contrasting findings also exist. For instance, memory impairment was found to be the most common domain affected in PD-MCI in some studies [5, 11]. Differences in cognitive measurement and definitions of PD-MCI may contribute to these discrepant findings.

Although patients with PD-MCI do not uniformly convert to dementia [15], studies have repeatedly demonstrated that by and large, patients with PD-MCI are at an increased risk of developing dementia, compared with cognitively intact PD patients [15–17]. It is still unclear which subtype is more likely to lead to dementia. Studies have suggested that posterior, rather than frontal cognitive function, predicted the development of dementia in PD [17–19]. It is possible that the posterior cognitive function is related to faster progression to dementia, while frontal-executive dysfunction is related to slower progression and with better prognosis [17, 20]. However, other studies have indicated that poor frontal/ executive performance but not memory impairment predicted later development of dementia [16, 21], and both executive dysfunction and memory deficits can predict dementia in PD [22]. In the studies where posterior cognitive functions (e.g., memory and visuospatial ability) were found to be associated with dementia progression [17, 20], frontal executive function was likely to be involved. This is because the memory and visuospatial tests used in the studies (e.g., the semantic fluency test and clock drawing test) also engage frontal executive function [6, 23]. Heterogeneous etiologies for PD-MCI patients could complicate tests of multiple cognitive functions (posterior vs. frontal/ executive). Since multiple neurodegenerative pathways can lead to MCI [24] and the subsequent conversion to dementia [25], the pathophysiology of PD-MCI is likely to be complex.

### Underlying neural mechanisms of PD-MCI

The clinical diagnostic criteria for PD-MCI by the MDS Task Force are built upon performance-based cognitive tests [8]. The cognitive manifestations of PD-MCI may be similar to those of MCI due to AD. To determine the specific physiological features of PD-MCI, neuroimaging methods can be useful. Some biomarkers, such as brain amyloid-beta (Aβ) protein deposition and cerebrospinal fluid tau/p-tau, hippocampal or medial temporal lobe atrophy on MRI, have repeatedly shown to predict the progression of MCI to dementia. As such, the inclusion of biomarkers in the diagnosis of MCI due to AD has been recommended by the National Institute on Aging-Alzheimer’s Association workgroups [26] to support the diagnosis. However, there has not been any reliable diagnostic neural biomarker for PD-MCI [27]. Although frontostriatal atrophy was found to predict dementia in PD-MCI [28], gray matter reduction in other regions, including the temporal, occipital, parietal, and supplementary motor areas, have also been implicated [29, 30]. In a large-sample study with drug naïve early PD patients, PD-MCI patients showed distributed cortical thinning in the temporal, parietal, frontal, and occipital areas, compared with healthy controls, and in the right inferior temporal cortex, compared with PD patients with normal cognition [31]. These observations reflect the difficulty in identifying unique neural mechanisms of PD-MCI.

PD-MCI patients have a distinct pattern of brain atrophy compared with PD patients with normal cognition, but show a similar pattern to that of PD patients with dementia, characterized by hippocampal, prefrontal, occipital, and parietal brain atrophy [32]. Longitudinal study further showed an AD-like pattern of brain atrophy (mainly in the hippocampus, medial temporal lobe and parietal–temporal cortex) to be associated with progression of cognitive decline and also predicted cognitive impairment in PD patients without dementia-level severity of cognitive impairment [33]. Other studies similarly showed hippocampal atrophy in PD patients with MCI or dementia [27, 34]. Given that hippocampal atrophy is the most established biomarker of AD pathology [35, 36], the observation of hippocampal structural alterations in the early stage of cognitive impairment of PD suggests an overlap with PD-MCI. Therefore, it is possible that PD-MCI might be due to the development of AD pathology among PD patients.

In addition to AD pathology, Lewy body (LB) disease has been associated with PD-MCI. Previous studies have demonstrated diffuse LB disease presentations in the transenthorhinal and entorhinal cortices, hippocampus, and some other limbic cortex as well as neocortex among PD patients [37, 38] and staging of pathological changes in LBs was the strongest correlate of the rate of cognitive decline in PD [37]. A recent study showed that attention impairment (a manifestation of frontal cognitive deficits) to be the most significant cognitive symptom of patients with LB pathology together with other non-motor symptoms, such as REM sleep behavior disorder and parkinsonism, that are commonly observed in PD [39]. Further, the pattern of gray matter volume loss in PD resembles more closely the pattern of atrophy observed in LB than in AD [34], suggesting that both entities may be part of a common spectrum [40]. α-Synuclein (α-syn) is a primary component of the LB bodies and has been implicated in the pathogenesis of PD by mounting evidence [41, 42]. Prior work has demonstrated that cerebrospinal fluid α-syn predicted cognitive decline in PD [43]. Finding from a recent work based on a publicly available database, the Parkinson’s Progression Markers Initiative (PPMI), indicated that amyloid-β (Aβ) alone and its interaction with α-syn contributed to cognitive decline and gray matter atrophy in the medial temporal, frontal, and brainstem structures in early PD [44]. In another study also using the PPMI data, the authors similarly noted that baseline Aβ level significantly predicted cognitive impairment at 2-year follow-up; furthermore, other biomarkers, including dopamine deficiency in the caudate and putaminal asymmetry, were also predictive of subsequent cognitive decline [45]. These findings suggest that cognitive impairment in PD may be primarily driven by Alzheimer-like amyloid pathology and secondarily by the synergic interaction between Aβ and α-syn. Of note, since these studies were launched before the MDS Task Force established the diagnostic criteria of PD-MCI [6], findings of cognitive impairment in these two studies did not necessarily follow the current MDS-based PD-MCI criteria. Future studies should validate the robustness of these biomarkers in predicting MDS-defined PD-MCI.

Although which subtype (posterior/ amnestic or frontal/ nonamnestic) of MCI is more associated with the development of dementia in PD is an ongoing debate, numerous studies have demonstrated an overlap between AD and LB pathology in PD-MCI. Hence, it has been proposed that a synergistic effect between LB and AD pathologies drive cognitive decline in PD [46, 47].

### Contributions of neuropsychiatric comorbidities to PD-MCI

Similar to cognitive impairment, neuropsychiatric disturbances, such as depression, anxiety, and apathy, are common non-motor symptoms in PD [48] and can appear in the early stage of disease [49]. Despite high prevalence of neuropsychiatric disturbances in PD, it has been suggested that conventional diagnostic criteria based on the Diagnostic and Statistical Manual of Mental Disorders (DSM) may not be suitable for PD patients. This is due to the difficulty in differentiating motor-related symptoms from mood-related somatic symptoms (e.g., fatigue and motor slowing) [50]. While the clinical definitions of depression, anxiety, and apathy specific to PD patients remain to be validated, the relationships between these neuropsychiatric disturbances and PD-MCI have received little attention. Nevertheless, limited evidence has shown that PD patients with depressive symptoms had significantly more severe cognitive deficits than patients with depression alone and healthy controls [11, 51]. Anxiety also shows negative correlations with processing speed, executive function, episodic memory, and language in some PD patients [52, 53], while apathy compromises attention and executive functions [53, 54].

Among individuals without PD symptoms, growing evidence has shown that neuropsychiatric symptoms may precede the prodromal stages of dementia (e.g., MCI) and increase the risk for conversion from minor neurocognitive disorders to major neurocognitive disorders [55]. A previous community-based study showed significantly higher rates of mood symptoms, including apathy, agitation, anxiety, irritability and depression, among elderly adults with MCI, compared to those with normal cognition [56]; in addition, mood symptoms increased the risk of subsequent development of MCI [57]. Although the prevalence of depression in individuals with MCI substantially varies across hospital-based and population-based studies, previous works indicated that depressive symptoms may be an early manifestation of dementia and share some neuropathological features with MCI or dementia (such as volume loss in the hippocampus) [58]. Similarly, anxiety has been shown to be associated with cognitive decline [59] in the elderly populations [60] and amyloid deposition in the brain, particularly in the frontal lobe and in older adults with MCI [61]. One study found that MCI patients with greater apathy had higher risk of developing dementia at one-year follow-up [62]. Another study showed that disconnection between the prefrontal cortex and mediodorsal and anterior thalamic nuclei might represent the neural substrates for apathy in MCI [63].

**Fig. 1:**
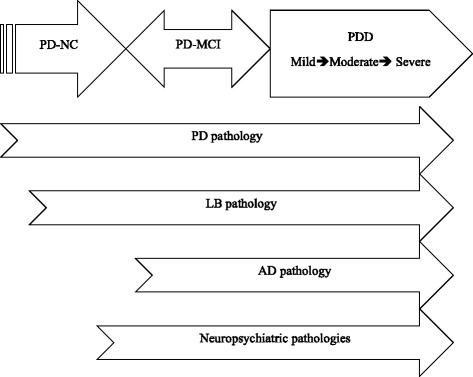
A schematic diagram showing the contributions of multiple pathologies to cognitive decline in Parkinson’s Disease (PD). Assuming dementia is the end point, some PD patients with mild cognitive impairment (PD-MCI) may progress to dementia (PDD), while some PD-MCI patients may revert to normal cognition (PD-NC) or stay with PD-MCI. Within the degenerative process, Alzheimer’s disease (AD), Lewy bodies (LB), and neuropsychiatric pathologies may insidiously develop alongside the existing PD pathology to worsen cognition

Based on the evidence of the link between neuropsychiatric disturbances and MCI in older individuals without PD symptoms, it seems plausible that depression, anxiety, and apathy may follow other neuropathologies that are not necessarily related to dopaminergic deficiency in the development of MCI or dementia. If this is the case, the underlying pathology of PD-MCI might be, at least in part, related to the evolving neuropsychiatric pathologies, rather than a specific manifestation of dopaminergic deficits or a collateral damage of PD pathology. Furthermore, patients with PD-MCI often suffer frontal-related cognitive dysfunction [12], which is commonly observed in patients with depression, anxiety, or apathy but without PD [59, 60, 64], thereby suggesting the shared neuropathogenesis. In such a case, neuropsychiatric pathogeneses may to some extent contribute to PD-MCI. Nevertheless, the specific role of neuropsychiatric pathologies in the development of MCI in PD would demand more future research endeavor to clarify. Figure 1 presents a schematic diagram of the potential pathological influence on the progression of cognitive impairment in PD.

### Practical considerations

In principle, it is of clinical importance to recognize PD patients at high risk for MCI. While multiple biomarkers of PD-MCI have been reported previously, caution is needed when one applies the biomarkers to identifying high-risk PD patients. Research in normal aging and AD has reported that a lack of direct relationships between the degree of brain pathology and the clinical presentations of the pathology [65]. Factors relating to life experiences, including education, occupational exposure, and leisure activities, may protect one from developing dementia and slow down cognitive decline. The combination of these factors has been termed *cognitive reserve* [66]. Despite that the buffering effects of cognitive reserve have been well characterized in normal aging, AD-related MCI, and AD [67–69], less attention has been paid to understanding the role of cognitive reserve in PD-related cognitive decline, especially in PD-MCI. A recent meta-analysis study revealed that cognitive reserve, specifically higher education, was associated with better cognitive performance and a reduced rate of cognitive decline in PD [70]. In addition, cognitive reserve may exert a protective effect on longitudinal cognitive decline and alleviate the impact of cortical Aβ accumulation in PD [71, 72]. Table 1 summarizes the existing studies on the relationship between cognitive reserve and cognitive decline in PD. Based on the findings from prior studies (Table 1), cognitive reserve or other unique life experiences likely influence the progression from MCI to dementia and may mask or alter the underlying neuropathology. Nevertheless, how cognitive research modulates cognitive decline in different PD-MCI subtypes and in the presence of biomarkers, such as Aβ and LB, remains unclear.

**Table 1:** Summary of studies on cognitive reserve in PD

Authors	Participants	CR measure	Outcome measure	Key findings
Sánchez et al. (2002)[72]	33 PDs 46 HCs	Education, occupation, and premorbid IQ	MMSE, IQ, memory, attention, language, visuospatial ability, and EF	• PDs with higher CR > PDs with lower CR: MMSE, verbal IQ, EF, memory, language, and visuospatial ability • PDs with higher CR= HCs with low CR: all tests, except one of the EF tests
Hindle et al. (2014)[69]^*a*^	PDs and HCs from 34 studies	Education	Global cognition, MCI, EF, attention, visuospatial ability, memory	• (+) corr between education and all the outcome measures • (-) corr between education and reduced cognitive decline • No corr between education and the onset of dementia
Hindle et al. (2015) [73]	57 monolingual English PDs 46 bilingual English PDs	Bilingualism	EF	• Bilingualism did not affect EF performance
Lucero et al. (2015)[70]	155 PDs	Education	MMSE, CDR, CDR-SB	• No corr between β-amyloid deposition and the cognition (MMSE & CDR) in high-education group • (+) corr between β-amyloid deposition and worse cognition (MMSE, CDR) in low-education group
Hindle et al. (2016)[71]	525 PDs	Education, SES, social engagement	Global cognition CDR	• (+) corr between education, SES, social engagement and global cognition at baseline and follow-up (+) corr between age and low social engagement and the risk of dementia
Hindle et al. (2017) [74]	69 non-demented PDs	Lifelong cognitive lifestyle (education, occupation, social engagement)	EF	• (+) corr between lifetime cognitive lifestyle and EF • No difference in executive function between high and low CR groups
Rouillard et al. (2017) [75]	49 PDs 47 HCs	Education, occupation, leisure and physical activities	Global cognition, episodic memory, visuospatial ability, attention, processing speed, and EF	• In HCs, (+) corr between CR and cognition • In PDs, education and occupation contributed to better cognition, but to a lesser extent than in HCs. • CR modulated the relationship between cognition and brain atrophy in PDs with less brain atrophy

^*a*^: a meta-analysis study, *(+) corr* positive correlation, *(-) corr* negative correlation, *CDR* Clinical Dementia Rating, *CDR-SB* Clinical Dementia Rating-sum of boxes, *CR* cognitive reserve, *EF* executive function, *HCs* healthy controls, *IQ* intelligence quotient, *MMSE* Mini-Mental State Exam; No corr: no significant correlation, *PD-NCs* cognitively normal PD patients; *PDDs* demented PD patients, *SES* socio-economic status

### Limitations of current studies

Increasing attempts have been made to examine the pathogenesis and clinical characteristics of PD-MCI. However, published studies have several limitations. First, variable definitions of PD-MCI and neuropsychological batteries used by different research teams may account for different cognitive profiles (posterior cognitive vs. frontal/ executive dysfunctions) found in PD-MCI studies. Second, most studies fail to take AD or LB pathology into account. Therefore, findings of medial temporal atrophy (e.g., hippocampal gray matter decreases) in PD-MCI could be due to the accumulation of AD pathology (e.g., Aβ). Compared with PD patients, LB pathology can present very similar cognitive profiles in patients with LB but without PD. However, given more similarities than differences between LB type dementia and PD type dementia [40], it might be challenging for future research to determine the relative contribution of LB pathology in PD-MCI based on clinical/behavioral presentations. Third, while high prevalence of neuropsychiatric comorbidities in PD and their impact on cognitive degeneration have been repeatedly highlighted, the majority of studies on PD-MCI did not consider the effects of these comorbidities on cognition, or on the conversion to dementia. Finally, individual life experience indicated by cognitive reserve may to some extent alter the odds of MCI onset and cognitive decline, but has not properly been considered in the search for a better understanding of the neuropathology of PD-MCI.

## Conclusions

PD-MCI is a common clinical condition. Understanding its pathology and clinical features is important for early intervention before the onset of dementia. In the past, variable definitions and differences in neuropsychological batteries generated divergent results of the affected cognitive patterns. The introduction of PD-MCI criteria by the MDS Task Force provides a more uniform system for defining and measuring PD-MCI and may improve the validity of future research. PD-MCI is likely to be heterogeneous since it can coexist with Alzheimer’s disease and/or LB pathologies in PD (Fig. 1) [40]. Neuropsychiatric disturbances, such as depression, anxiety and apathy, are associated with PD with or without MCI. The overlap of cognitive impairment across different neurodegenerative diseases suggests a complex pathophysiology. As such, PD-MCI is likely to manifest a mixture of multiple pathologies, rather than being a distinct pathologic entity. Differentiating MCI from other organic symptoms in PD can facilitate novel therapeutic strategies. Finally, while focusing on identifying biomarkers to elucidate the underlying neuropathology, individual life experiences, such as cognitive reserve, should also be taken into account.

## Abbreviations

AD: Alzheimer’s disease
Aβ: Amyloid-beta
DSM: Diagnostic and statistical manual of mental disorders
LB: Lewy body
MDS: Movement disorders society
PD: Parkinson’s disease
PD-MCI: Mild cognitive impairment in Parkinson’s disease
PPMI: Parkinson’s progression markers initiative
α-syn: α-synuclein
